# Diet Therapy in Eosinophilic Esophagitis. Focus on a Personalized Approach

**DOI:** 10.3389/fped.2021.820192

**Published:** 2022-01-20

**Authors:** Martina Votto, Maria De Filippo, Marco Vincenzo Lenti, Carlo Maria Rossi, Antonio Di Sabatino, Gian Luigi Marseglia, Amelia Licari

**Affiliations:** ^1^Department of Clinical, Surgical, Diagnostic and Pediatric Sciences, University of Pavia, Pavia, Italy; ^2^Department of Internal Medicine, Fondazione IRCCS Policlinico San Matteo, University of Pavia, Pavia, Italy; ^3^Pediatric Clinic, Fondazione IRCCS Policlinico San Matteo, Pavia, Italy

**Keywords:** eosinophilic esophagitis, diet, food allergens, food-reintroduction, personalized therapy, multidisciplinary approach, phenotype, endotype

## Abstract

Eosinophilic esophagitis (EoE) is a chronic allergic disease defined by a marked eosinophilic inflammation and symptoms of esophageal dysfunction. EoE is a heterogeneous disease and severely impacts the quality of life of affected patients. The current therapeutic management of EoE is based on two cornerstones: medication and diet therapy, both effective but limited by several critical issues. The choice of one or the other therapy might depend on the different disease phenotypes (allergic vs. non-allergic, inflammatory vs. fibro-stenotic), patient's age (adult vs. childhood-onset), food habits, patient/family preference, and familiar financial resource. Diet therapy is a successful treatment but limited by low patient adherence, the need for several endoscopies, food restrictions, psychosocial impact, and potential nutritional deficiencies. All these limitations could be effectively overcome with multidisciplinary and personalized management. This review summarizes the most recent evidence on the dietary elimination approaches and will provide a practical guide to clinicians in managing and implementing dietary therapy for patients with EoE.

## Introduction

Eosinophilic esophagitis (EoE) is the most characterized eosinophilic gastrointestinal disorder (EGID) and is a chronic/remittent allergic disease, defined by a marked eosinophilic inflammation and symptoms of esophageal dysfunction (1, 2). Currently, the diagnosis of EoE requires the presence of more than 15 eosinophils per high power field (eos/HPF) in the endoscopically obtained esophageal biopsies in patients with suspicious symptoms (1, 2).

It is estimated that EoE affects about 0.5-1/1,000 patients in the USA, varying widely across the different Countries and mostly prevailing in Caucasian patients and male sex (3). However, in the last 20 years, several epidemiological studies showed a significant increase in the epidemiology of EGIDs, partially related to improved medical awareness and knowledge through modern diagnostic instruments (4–6). It was also postulated that changes in environmental factors may have contributed to the significant increase in EoE epidemiology (7). Recently, Navarro et al. found that the pooled prevalence of EoE is 34.4 cases/100,000 inhabitants and is higher for adults than for children (42.2/100,000 vs. 34/100,000) (5). The pooled incidence rate was 6.6/100,000 people per year in children and 7.7/100,000 in adults (5).

**Figure 1:**
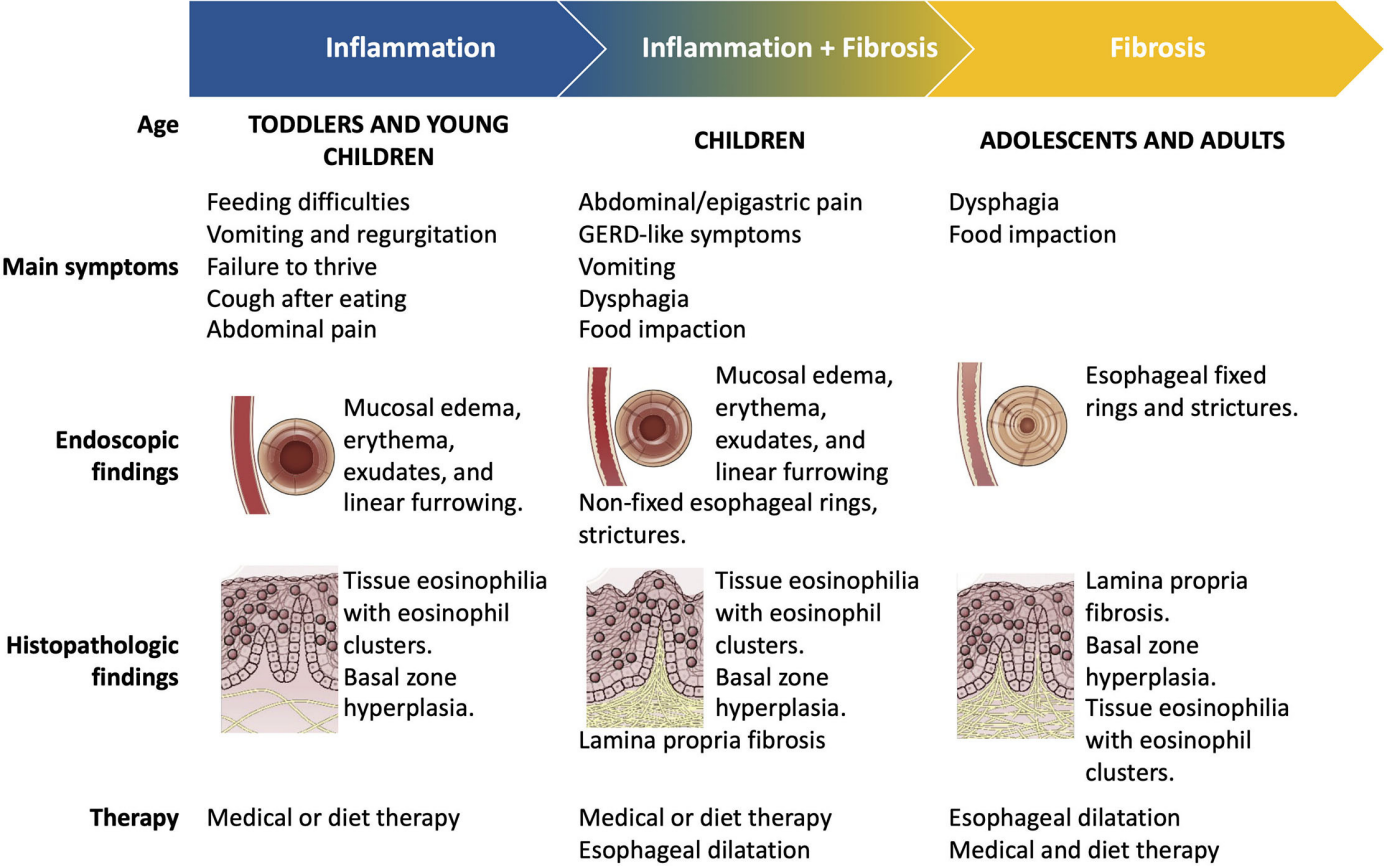
Natural history and clinical heterogeneity of eosinophilic esophagitis.

Genome-wide association studies have identified multiple susceptibility genes associated with EoE risk and a complex model of disease inheritance. EoE is a multifactorial disease typically characterized by a type 2 (T2) inflammation (8). The impaired epithelial barrier function plays a pivotal role in the pathophysiology of EoE, inducing the release of alarmins (thymic stromal lymphopoietin, IL-15, IL-33), which then activates the type 2 innate lymphoid cells (ILC2) and basophils. The subsequent release of IL-4, IL-5, and IL-13 recruits and expands the eosinophilic inflammation. The consequences of this sustained inflammation include tissue remodeling and esophageal dysfunction. Esophageal fibrosis begins in the early phases of the disease course, initially involving the *lamina propria* (6). Fibrosis has been found in 57–88% of young patients and children and 89% of adult patients with EoE (9). However, the increased esophageal stiffness, due to subepithelial fibrosis and muscular hypertrophy, clinically occurs with food impaction and dysphagia, symptoms that are typically reported by adult patients (Figure 1) (9). Although the pathogenesis is not entirely understood and is likely non-IgE-mediated, food allergens are known to trigger EoE, stimulating the already dysregulated immune cells through the impaired esophageal epithelial barrier (10, 11). Most patients with EoE are allergic to 1–3 foods that trigger esophageal inflammation, according to Koch's postulate (12). Esophageal inflammation is resolved once the food(s) is removed from the diet, and reproducibility reactivates it when the culprit allergen(s) is reintroduced (10, 12–14). Recent and conflicting studies have also supported the potential role of aeroallergens in the pathogenesis of EoE, with evidence mostly limited to case series and case reports (8, 15).

Since EoE was first recognized as a distinct clinical entity in the mid-1990's, several signs of progress were achieved. However, there are diagnostic and therapeutic aspects that should be investigated, and one of these concerns the diet therapy and nutritional assessment of patients with EoE. To date, most data on nutritional management came from the single center's experience rather than comparative clinical trials. This review summarizes the most recent evidence on the dietary elimination approaches and will provide a practical guide to clinicians in managing and implementing dietary therapy for patients with EoE.

## Clinical Features and Heterogeneity of EOE

EoE is a heterogeneous disease with variable symptoms and severity, comorbidities (atopic vs. non-atopic), treatment response, and natural history. Moreover, EoE severely impacts both adults and children's quality of life (QoL) (16, 17). Notably, EoE symptoms vary with age (1). Toddler and young children generally experienced food refusal, feeding difficulties, and recurrent vomiting and/or regurgitation. On the contrary, school-aged children reported abdominal/epigastric pain, refractory gastroesophageal reflux, whereas adolescents and adults present dysphagia and food impaction. Symptoms, endoscopic and histological findings, and response to treatments reflect the typical evolution of the EoE inflammation through time, as reported in Figure 1. In this context, different clinical patterns or phenotypes have been identified (16). The “inflammatory” pattern is generally observed in childhood and is defined by the endoscopic evidence of edema, erythema, linear furrowing, and the prevalent eosinophilic esophageal inflammation in histologic samples (16, 18, 19). On the other hand, the “fibro-stenotic” and “fibrotic” phenotypes primarily affect adolescents and adults with dysphagia and food impaction (16, 18–20). These phenotypes are endoscopically characterized by fixed esophageal rings and/or strictures resulting from tissue remodeling and esophageal fibrosis (16, 18–20). The clinical and histological heterogeneity might reflect and partially explain the heterogeneous response to available therapies (16). While diet and medical therapies may reduce tissue fibrosis in childhood, this remodeling process may persist despite the resolution of inflammation in adulthood (6). Recently, Shoda et al. identified three potential endotypes of EoE, using a machine learning approach to analyze histological, endoscopic, and molecular features of US patients with EoE. The first endotype (EoEe1) was recognized in 35% of the cohort and was mainly characterized by a minimal eosinophilic inflammation and steroid responsiveness. The EoEe2 endotype affected 29% of patients and showed a prevalent T2 inflammation, pediatric-onset, and a low steroid response. Finally, 36% of the EoE cohort (EoEe3) presented an adult-onset and structuring disease (16, 21). Therefore, according to this endotype classification, patients with the EoEe1 endotype might successfully be treated with diet and steroid therapy, and children with the EoEe2 endotype might benefit from the anti-T2 immune agents (i.e., dupilumab) (22). Finally, adult patients with the EoEe3 endotype are more challenging to treat with available therapies; thus, esophageal dilatations are the only current solution to esophageal stenosis (22). In the future, a validated endo-phenotype classification of EoE will provide better disease management and aim physicians to develop a personalized medicine using targeted treatments.

## How to Manage EOE

The current therapeutic management of EoE is based on two cornerstones: the medication (proton pump inhibitors [*PPIs*] and topical corticosteroids) and diet therapy, both effective but limited by different critical issues (2, 17, 18). Patients with EoE should be maintained on monotherapy when effective (2, 23). However, if monotherapy fails or loses its efficacy, a combination therapy (diet + topical steroid) may be indicated (24). Although not already approved, biological therapy with dupilumab showed promising results in adults with EoE, improving symptoms, esophageal inflammation, and distensibility (25).

When correctly administered (1 mg/kg/day, twice daily), PPIs are effective in about 50% of children with EoE. The long-term effectiveness of PPIs is still debated and might be related to specific genetic polymorphisms (26, 27). However, disease remission might appear more sustained in patients with the inflammatory phenotype than those with the fibro-stenotic or stenotic phenotype (19, 26, 27). Therefore, as widely reported, PPI response is not homogeneous and prolonged in all patients (27).

Current formulations of topical corticosteroids have not yet been approved by the Food and Drug Administration (FDA) (27). However, in 2017, the European Medicines Agency (EMA) authorized orodispersible budesonide for adults with EoE (https://www.ema.europa.eu/en/documents/assessment-report/jorveza-epar-public-assessment-report_en.pdf). Slurry budesonide and swallowed fluticasone are both effective to induce EoE remission. However, their long-term use is compromised by patient adherence and side effects (19, 27). Although topical corticosteroids are generally safe and well-tolerated, long-term administration is complicated mainly by esophageal candidiasis in 1–3% of patients (19, 27). Moreover, there have been sporadic reports of decreased cortisol levels, minor anthropometric growth changes, and low bone mineral density; thus, physicians may consider periodic monitoring for growth, adrenal, and bone metabolism (27). When complete remission is achieved, topical corticosteroid treatment should be administered at the minimal effective dosage to reduce the risk of potential long-term side effects. On the other hand, a brief cycle of oral/systemic corticosteroid is also suggested for controlling refractory esophageal inflammation (28).

In 1995, Kelly et al. successfully demonstrated the efficacy of the exclusive aminoacid-based formula diet in children with EoE (29). Since this attempt, several studies have evaluated the therapeutic role of elimination diets. Three main dietary approaches, such as the elemental, empiric, and allergy test-directed elimination diets, have been proposed with variable efficacy rates and specific advantages and disadvantages (Table 1) (2, 28). Although the therapeutic choice mainly depends on clinician experience and patient's needs, several clinical aspects must be considered before prescribing a diet therapy, especially in children.

**Table 1:** Diet therapies of eosinophilic esophagitis.

**Diets**	**Specific recommendation**	**Results**
Elemental diet	Elemental formula	Adults and children ~ 90%
**Elimination diet**		
*6-food*	Cow's milk, wheat, eggs, soy/legumes, seafood, nuts	Adults 52–70% Children 74%
*4-food*	Cow's milk, wheat, eggs, soy/legumes	Adults 52–70% Children 74%
*2-food*	Cow's milk, wheat	Adults and children 43%
*1-food*	Cow's milk	Adults and children 44–70%

*IgE, immunoglobulin E*.

## Diet Therapy

### What Clinicians Should Know Before Prescribing a Diet Therapy

According to international guidelines, the diet approach is considered the first-line treatment of EoE and is as effective as medication therapy (2, 28). It is widely demonstrated that foods are the primary triggers of EoE; indeed, food elimination diets (FEDs) have demonstrated complete remission of EoE, with higher rates (>90%) in patients treated with elemental diet than empirical FEDs and test directed diets (12). However, FEDs are challenging and are not risk-free. Patients on diet therapy may potentially develop nutritional deficiencies, eating disorders and experience a low QoL and high psychological impacts. Before prescribing a FED, allergists and gastroenterologists should consider several clinical aspects, such as (1) disease-severity and patient's nutritional status, (2) presence of maladaptive feeding behaviors or/and food allergies, (3) family and patient preferences, and (4) financial resources (27). Then, clinicians should widely explain to patients and their families the advantages and disadvantages of diets to choose judiciously (7, 27). Children and adults, candidates for diet therapy, should also be informed of the need to undergo several endoscopic and clinical evaluations to confirm or assess disease remission (2, 28). Patients and parents of children with EoE should know that more restrictive diet therapies (elemental and empirical FED) may be expensive and alternative foods may be often found only in specialty stores (30). On the other hand, clinicians should guarantee a strict follow-up with upper GI endoscopy to evaluate the remission 6–12 weeks after diet beginning and each food reintroduction (2, 28). Moreover, physicians should consider patients' food habits, such as eating at home/work or school canteen, reliance on pre-prepared foods, and cultural issues (12).

At baseline, patients with active EoE are generally not malnourished (31). However, toddlers and young children may present growth failure and feeding issues that are not a contraindication for an elimination diet after a comprehensive assessment of the nutritional status (32). As reported in different pediatric studies, a significant proportion of children with EoE has other coexisting allergic diseases, including multiple IgE-mediated food allergies (33–35). These patients generally are not the best candidates for FEDs, as the extensive food restrictions may compromise patient's compliance and negatively impact on QoL (14).

### Elemental Diet

The elemental diet consists in removing all foods. Thus, patients are exclusively fed with an aminoacid-based formula for at least 6 weeks (2, 28, 36). The elemental diet is the most effective treatment, and several studies reported high complete remission rates in children and adults with active EoE (37). EoE patients treated with the elemental diet experienced a significant reduction in their symptoms and achieved complete histologic remission in 90 and 94% of pediatric and adult cases, respectively (Table 1). Moreover, the highest efficacy rates are primarily observed in patients with a non-stricturing phenotype (27, 38–41).

The elemental diet is a fundamental therapeutic option, especially in severe EoE cases. However, the elemental diet is not the first-line approach for its limitations in most cases (12). Elemental diet is often proposed as rescue therapy or temporary solution in adults and adolescents with refractory EoE when all other treatments alone or in combination have failed (12, 27). In toddlers or young children with active EoE complicated by failure to thrive, the elemental diet is generally considered a valid and useful therapeutic option with the highest patient compliance (12, 27). In severe disease or when large volumes of the aminoacid-based formula are required to meet the caloric needs and restore the good nutritional status, nasal-gastric (NG) or gastric (G) tube feeding is a temporary solution (42). These interventions should be discouraged in the long-term treatment, especially in children with feeding disorders, because they are often fraught with difficult solid food oral reintroduction and progressive feeding skills regression (12). In children with multiple food triggers and subsequent high diet restrictions, elemental formulas can also be used as supplements of protein and energy necessary for adequate growth and puberty spurt (12).

Although the elemental diet can induce a rapid disease-remission in only 2 weeks, several disadvantages limit its adherence (Table 2) (43). The poor palatability, highly restrictive nature, costs, and psychosocial isolation are the main reasons for treatment discontinuation and low compliance (12, 17, 27). To remedy these issues, the elemental diet is often modified, introducing one or two less allergenic foods (generally vegetables or fruits) in addition to the aminoacid-based formula (12, 27). Moreover, elemental formulas are also available in flavored and unflavored formulations to address patient taste and preferences (12). Pediatric elemental formulas are nutritionally complete but do not contain dietary fiber. Thus, fiber supplements (free of known allergens) should be prescribed in patients who develop or are more prone to constipation (12).

**Table 2:** Advantages and disadvantages of elemental diet.

**Advantages**	**Disadvantages**
• Rapid and complete remission in 2 weeks • Better acceptance in young children and toddlers • Rescue therapy or temporary solution in adults with non-stricturing EoE • Pediatric formulas are almost nutritionally complete • Nutritional supplement	• Poor palatability and low patient's compliance • The administration through NG or G-tube may induce feeding skills regression • High cost and insurance coverage • Less effective in stricturing EoE

*EoE, eosinophilic esophagitis; G, gastric; NG, nasal gastric*.

### Food Elimination Diets

#### Empirical FED

**Figure 2:**
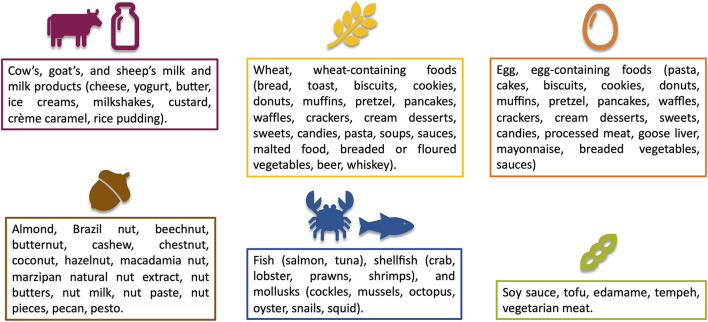
Most allergenic groups of foods that trigger eosinophilic esophagitis.

In general, more foods are eliminated from the diet, more likely the remission is achieved at the first endoscopy. FED is the most widely used diet treatment for EoE. The first proposed FED was founded on avoiding the six most common food-triggers of EoE in the Western diet, such as milk, wheat, egg, soy/legumes, peanut/tree nuts, and seafood/fish (Figure 2) (44). Patients should be advised that all these foods should be avoided both in fresh and backed forms (12). The 6-FED effectively induces histologic remission in about 74% of children and 70% of adults with EoE (Table 1) (45). Studies assessing the efficacy of 6-FED have been fundamental to find that the most common food triggers are cow's milk (up to 85% of the pediatric cases), followed by wheat/gluten (up to 60%), egg, and soy/legumes with geographic variations, primarily due to the different food cultures (37). Consequently, nuts and fish/seafood rarely trigger EoE. Therefore, most of the patients who histologically recover with 6-FED were allergic to only 1–3 foods (19).

Although 6-FED is less restrictive than the elemental diet, it still can be challenging to avoid all the six groups of foods. Several drawbacks limit the adherence to 6-FED due to the high level of dietary restriction and the need of frequent upper GI endoscopies to identify the culprit food(s) (12). For these reasons, 6-FED is generally not considered the ideal therapeutic approach in most EoE patients. Therefore, subsequent studies proposed and assessed the utility of less restrictive FEDs that consisted of avoiding the most common food triggers. 4-FED (milk, wheat, egg, and soy/legumes-free diet) induced histologic remission in 64 and 54% of children and adults, respectively (46, 47). In studies evaluating the efficacy of 4-FED, milk and wheat were the most common triggers of EoE (12). Children and adults avoiding these two foods (2-FED) achieved complete remission in 40 and 44% of cases, respectively. The elimination of cow's milk (1-FED) demonstrated disease-remission rates of 44–51% in pediatric patients (27). In a recent systematic review with meta-analysis, the overall efficacy of a milk-free diet was about 70% (45).

**Figure 3:**
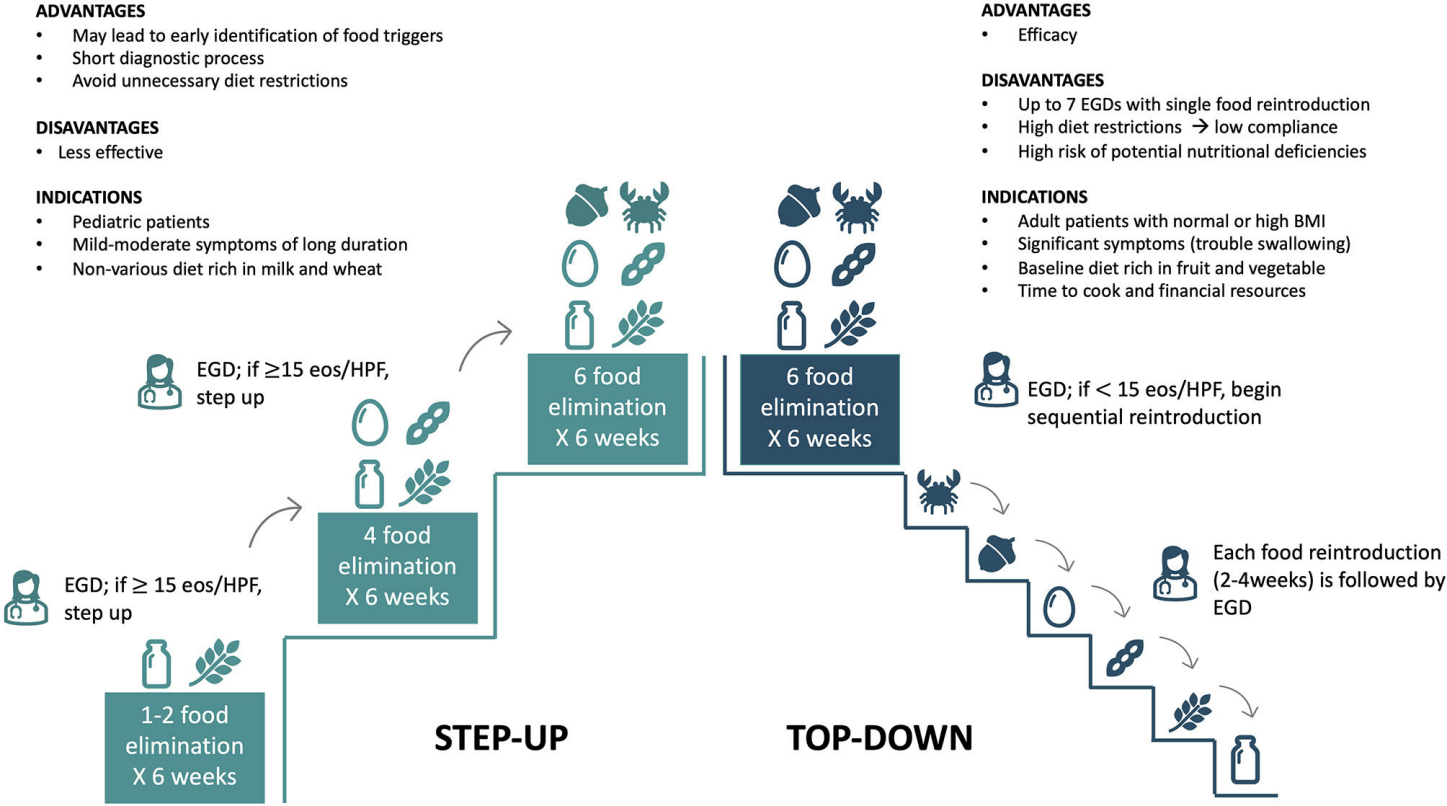
Top-down and step-up approaches: indications, advantages, and disadvantages. EGD, Esophagogastroduodenoscopy.

There are two strategies for avoiding foods in FED with different indications, strengths, and weaknesses (Figure 3) (12, 27). FED can be managed with a *top-down approach* removing milk, wheat, egg, soy/legumes, peanut/tree nuts, and seafood/fish (6-FED) simultaneously. If disease-remission is achieved (<15 eos/HPF), the avoided foods can be sequentially added to the patient's diet, with clinical evaluations and esophageal-gastroduodenoscopy after each reintroduction, to identify the true allergenic trigger(s) (12, 22). Although more effective, this approach is limited by several endoscopic procedures (at least six), high diet restrictions, and potential nutritional deficiencies that may negatively impact patient and family compliance (12, 22). Moreover, a more restrictive diet requires high financial resources and time for buying and preparing alternative meals. For these reasons, the top-down approach is generally indicated in adult patients with severe esophageal symptoms limiting the normal feed (i.e., swallowing issues), adequate/high body mass index (BMI), and without nutritional deficiencies (12). Recently, Molina-Infante et al. tested a prospective *step-up approach* to empiric food elimination (13). The step-up approach consists of the initial elimination of one (1-FED) or two (2-FED) more common allergenic foods (milk and wheat) (13). If a complete remission is not achieved, diet is further restricted to a 4-FED and eventually to 6-FED (13). Although less effective, this dietary approach leads to faster and earlier identification of food triggers [one to four GI endoscopies to identify food trigger(s)] than the top-down approach, avoiding unnecessary diet restrictions (12, 13, 27). A step-up approach is generally preferred in children and adolescents with mild-moderate GI symptoms, a diet rich in milk and wheat, and signs of impaired growth or BMI (Figure 3).

#### Allergy-Test Directed Elimination Diet

EoE is a T2 immune-mediated disease, where IgEs do not have a specific pathogenetic role. Based on the results of skin prick tests and atopy patch tests, Spergel et al. reported that about 75% of children achieved a significant improvement in EoE symptoms and esophageal inflammation (48). However, subsequent studies found that atopy patch tests, skin prick tests, food-specific serum IgEs did not reliably predict food triggers and did not have a clear role in evaluating patients with EoE (49, 50). Moreover, a meta-analysis revealed that this diet approach induces histologic remission in 45.5% of patients, and efficacy rates were significantly lower in adults than in children (45). According to this evidence, current American and European guidelines do not recommend allergy test-based dietary elimination therapies (2, 28, 51).

## How to Manage Food Reintroduction and Long-Term Treatment?

**Figure 4:**
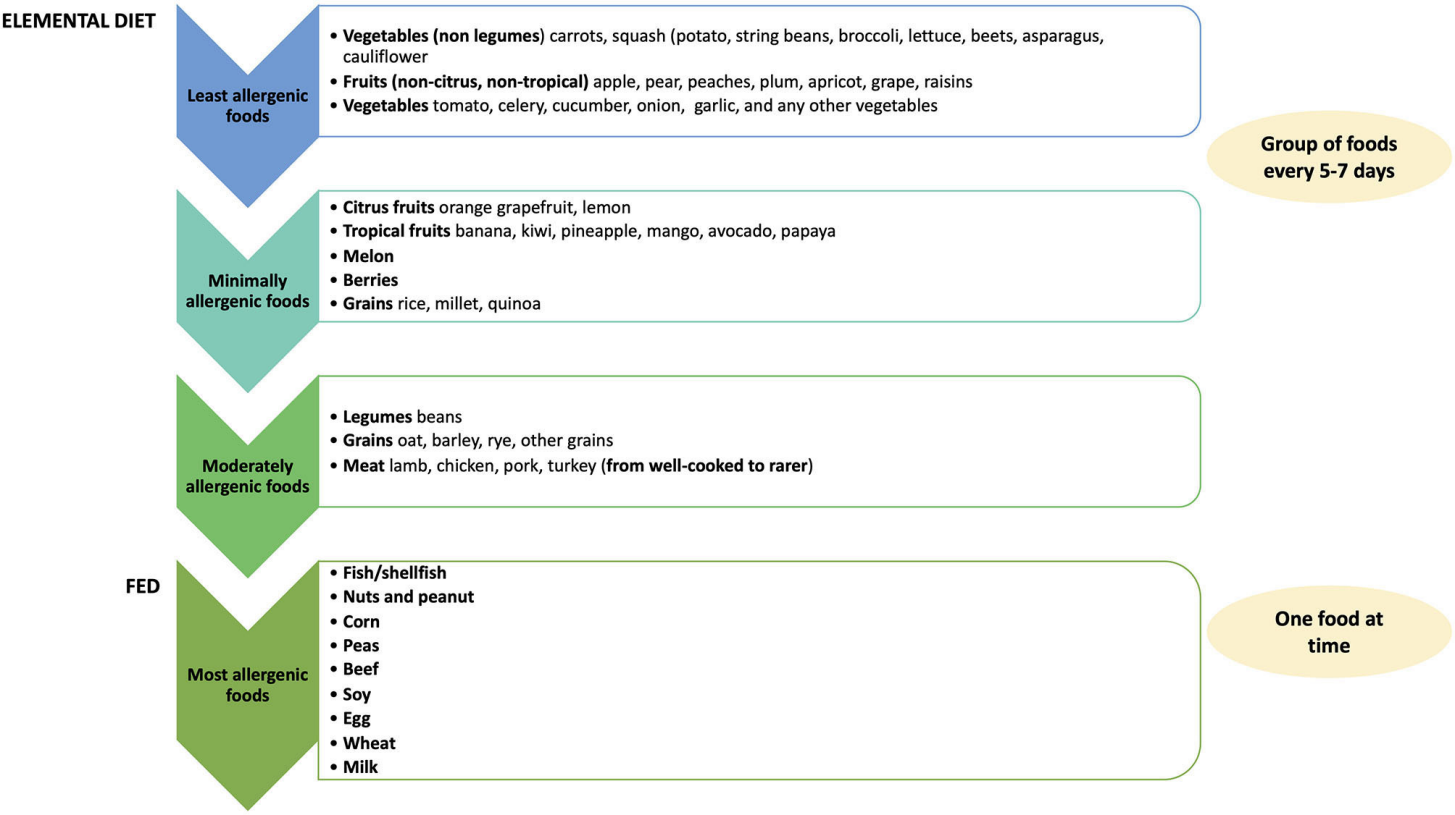
Food reintroduction.

When a FED (empirical food elimination or elemental diet) is implemented, the GI endoscopy should be performed after 6–12 weeks to assess the histologic remission (2, 30). Once clinical and histologic remission is achieved, a single food or food group is gradually reintroduced based on the specific diet approach. The endoscopy should be made after 4–6 weeks each reintroduction to confirm or exclude disease remission and before proceeding to other food reintroduction (2, 28, 43). Food reintroduction should start from the less allergenic foods (fruits and vegetables) to the most common food triggers (52). In patients treated with elemental diet or 6-FED, Cianferoni et al. recommended reintroducing the high-risk foods (milk, wheat, soy, and/or egg) one at a time, whereas medium-risk foods (legumes, seafood, nuts) may be re-administered at one time, and low-risk foods (fruit and vegetables) may be reintroduced in groups every 5–7 days (Figure 4) (12, 52). If symptoms do not recur after reintroducing 4–5 new foods from one group, endoscopy is performed 1–2 months later (52). On the contrary, if patients become symptomatic or relapse after reintroducing a specific food, that food is definitively excluded from the diet (52).

If EoE children achieve complete disease remission on a free-milk diet (1-FED), cow's milk and milk-containing products (included all mammalian milk and partially and extensively hydrolyzed formulas) should be removed from the diet. However, it is reported that some patients can tolerate baked milk products that may be tried in the diet followed by an upper GI endoscopy. Notably, children sensitized (positive food-specific IgEs or skin test) to previously tolerated foods removed from the diet because of EoE triggers, should be referred to a pediatric allergist before the reintroduction at home (12). As already reported in patients with atopic dermatitis, children with EoE may develop IgE-mediated immediate hypersensitivity to food previously identified as the causative agent for EoE (53, 54).

A significant group (~20%) of adults and children treated with an elimination diet do not respond to the dietary approach, even after mostly eaten trigger foods are removed, or a more restrictive 6-FED is implemented. In these cases, after assessing the patient's compliance, combination therapy with FED + PPI is generally recommended, or clinicians may add a topical corticosteroid and gradually expand the diet, reintroducing foods (12).

Due to its chronic/remittent nature, EoE requires lifelong therapy (2, 28). Patients following a dietary regimen should be widely informed of the need for repeated follow-up endoscopies. Food reintroduction in patients treated with a 6-FED requires at least six endoscopies and several months to identify the culprit food(s). In children exclusively fed with the aminoacid-based formula, the food-reintroduction process is even longer and loaded by several endoscopies. Once the culprit food(s) is identified, the long-term diet therapy is only based on exclusively avoiding the food(s) responsible for esophageal inflammation (55). In adults, the strict avoidance of trigger food(s) maintains a complete remission (clinical and histologic remission) for up to 3 years (43, 56). Notably, the prolonged elimination of a food or a group of trigger foods might induce potential nutritional deficiency.

## Nutritional Considerations and Patient Education

**Figure 5:**
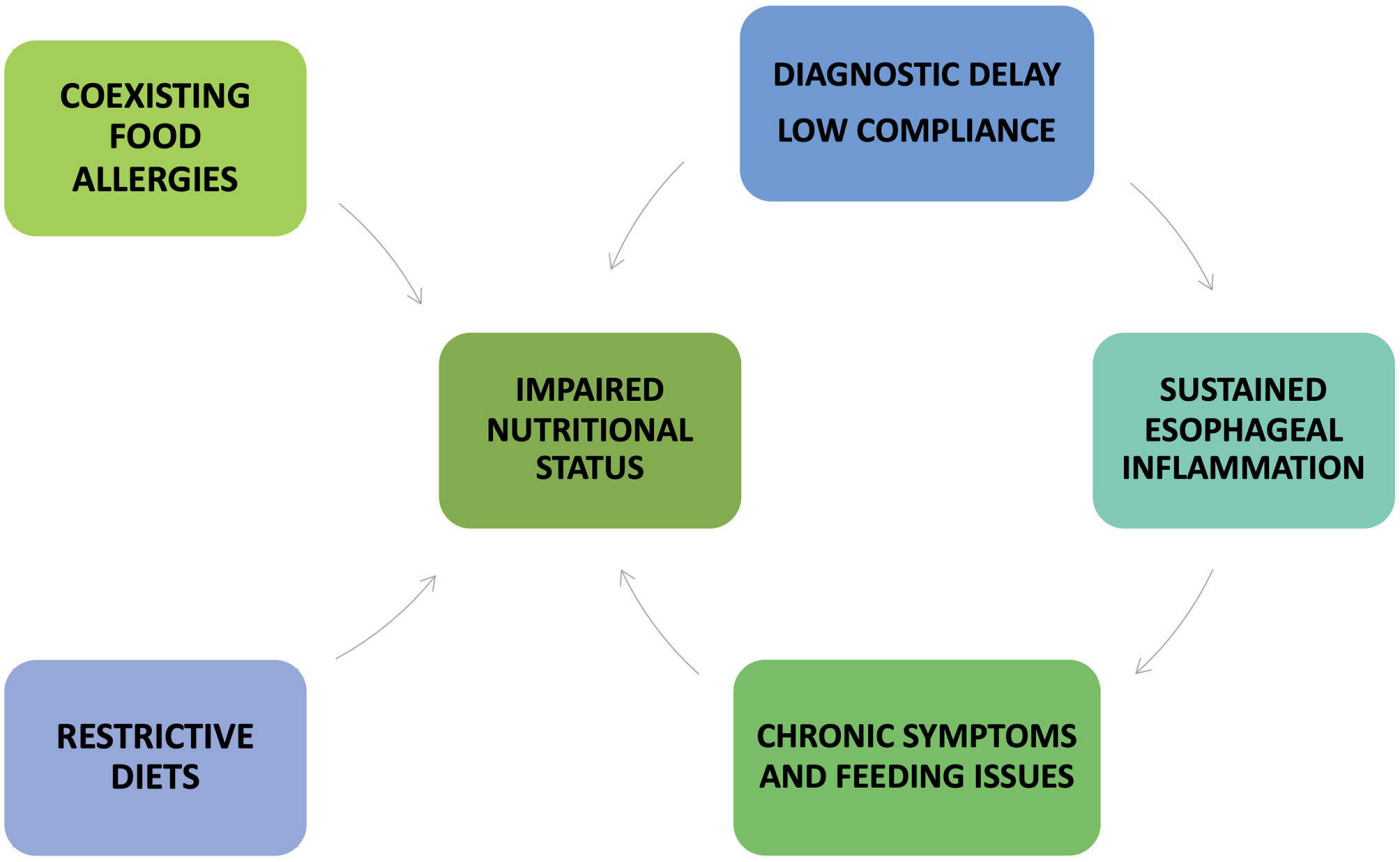
Factors that may negatively impact the nutritional status of patients with eosinophilic esophagitis.

Several factors may negatively impact the nutritional status of patients with EoE (Figure 5).

Firstly, children with EoE generally present symptoms that may limit the adequate nutritional intake, such as recurrent vomiting and regurgitation, abdominal pain, lack of appetite, low volume and/or poor variety food intake, grazing, and spitting food out (31). Patients with chronic esophageal inflammation develop compensative feeding habits (i.e., drinking a lot during meals, eating slowly, chewing carefully, cutting food into small pieces, lubricating foods with sauces or liquids), or avoiding some foods (meat, crusty bread, pills) (20). Moreover, young children fed for a long time with liquid formula do not engage masticatory muscles and are at increased risk of delayed onset of oral-motor skills (57).

Secondly, EoE is often delayed or misdiagnosed. It is reported that diagnostic delay mainly occurs in the first two decades of life and is more likely associated with tissue remodeling complications, such as esophageal rings and strictures, and further prolong the GI symptoms and feeding discomfort (58, 59).

Although occurring in pediatric patients, esophageal strictures generally complicate the disease course in adulthood since esophageal fibrosis becomes an irreversible process more challenging to treat with available therapies (58). Patients with previous food impaction episodes may have a high risk of developing anxiety and eating disorders, compromising the adequate nutrient intake (17, 60). Therefore, chronic GI symptoms, compensative feeding habits, eating disorders may all complicate the nutritional status of EoE patients, especially if they are children.

Thirdly, the coexistence of multiple (IgE and non-IgE mediated) food allergies might be a further reason for failure to thrive and undernutrition. On the other hand, long-term restrictive FEDs may compromise adequate micronutrient intake, although they do not appear to worsen child growth or BMI (31, 61). For these reasons, in children treated with 1- or 2-FED, regular clinical follow-up is recommended to identify early potential nutritional deficiency and growth impairment (Table 3).

**Table 3:** Nutritional assessment [Adapted from Cianferoni et al. (12)].

**Nutritional assessment**	**Parameters**	**Health care specialist**
Clinical history	Symptom onset Food-related symptoms Extraesophageal manifestations and comorbidities	Gastroenterologist Allergist Pediatrician
Anthropometric data	Weight Height BMI	Gastroenterologist Allergist Pediatrician Nutritionist
Patient diet and feeding habits	Breakfast, lunch, snacks, dinner (food diary) Food variety	Nutritionist
Identification of feeding issues	Description of a typical meal; food and texture preferences. Swallowing issues Delayed onset of oral-motor skills	Nutritionist
Identification of eating disorders, behavioral issues, and neurological diseases	Unmotivated weight loss Nervous anorexia Anxiety Depression Fear of eating in public Fear of food impaction Autism spectrum disorders	Psychologist
Coexisting allergic and non-allergic comorbidities	Gastroesophageal reflux diseases Coeliac disease Inflammatory bowel diseases Esophageal atresia IgE and non-IgE mediated food allergies Food intolerances Atopic dermatitis	Gastroenterologist Allergist Pediatrician
Biochemical assessment	Complete blood count Iron status (serum ferritin, iron, total iron-binding capacity, hemoglobin) Bone metabolism (calcium, phosphate, vitamin D, alkaline phosphatase)^*^ Micronutrient deficiency (folate, vitamin B12, zinc, selenium, electrolytes) Macronutrient deficiency (albumin, prealbumin, total protein, blood urea nitrogen, creatinine)	Gastroenterologist Allergist Pediatrician Nutritionist
Compliance to therapy	Follow-up EGD with biopsies Clinical scores	Gastroenterologist Allergist Pediatrician

* *No current guidelines exist on DEXA use in patients on a milk-free diet or topical steroid therapy (12)*.

*BMI, body mass index; EGD, esophageal-gastroduodenoscopy; IgE, immunoglobulin E*.

Finally, the low compliance to therapy is the main reason for therapeutic failure and persistent active inflammation (17).

Nutritionists have a crucial role in evaluating nutritional status (Table 3). A nutritionist should meticulously evaluate the diet of patients (i.e., veggie or lactose-free diets) to determine the degree of exposure to high-risk groups of foods and the potential nutritional and psychological effects of their elimination (62, 63). Before beginning a diet therapy and during the follow-up period, clinicians should periodically assess the nutritional status of patients and rule out the potential nutritional deficiency. Then, clinical (symptoms, comorbidities, feeding habits/disorders) and anthropometric data should be collected and carefully evaluated to address the best therapeutic choice.

EoE may appear with failure to thrive, one of the most described complications in young children (64, 65). Moreover, the risk of nutritional deficiency and impaired growth also increases with the restrictive nature of the diet and the number of removed foods. Vitamin D deficiency is widespread in Western Countries and is frequently found in patients with chronic inflammatory diseases, including allergic disorders (31). Although published studies are often conflicting, patients with EoE are at high risk of impaired bone metabolism and vitamin D deficiency due to the intrinsic nature of the esophageal inflammation, long-term treatment with topical corticosteroids, and FED (31). Iron deficiency anemia may be a consequence of selective diets. The fear of new food impaction episodes leads patients to voluntarily remove the culprit food (especially steak). If failure to thrive or nutritional deficiencies are suspected, biochemical tests (i.e., bone and iron metabolism, serum albumin, and prealbumin) should be performed. When a micro- or macronutrient deficiency is confirmed, nutritional supplements should be promptly provided (Table 4) (66).

**Table 4:** Nutritional deficiencies associated with food elimination and appropriate substitutions [Adapted from Bashaw et al. (66)].

	**Milk**	**Wheat**	**Egg**	**Soy**	**Nuts**	**Fish/shellfish**
**Macronutrient**						
Protein	X		X	X	X	X
Fat	X		X	X	X	X
Fiber		X			X	
**Micronutrient**						
Calcium	X			X		
Vitamin D	X		X			X
Iron		X		X		X
Zinc		X		X	X	X
Copper					X	X
Selenium		X	X		X	X
Vitamin A	X		X			
B1—Thiamin		X		X		
B2—Riboflavin	X	X		X		
B3—Niacin		X		X	X	
B5—Pantothenic acid	X		X			
B6—Pyridoxine		X		X		
B7—Biotin		X	X			
B9—Folate		X		X	X	
B12—Cobalamin	X		X			X
Iodine	X					X
**Substitutions**	Meats, legumes, whole grains, nuts, fortified foods, and beverages	Fortified foods, fruits, vegetables, other grains (barley, oat, rice, corn, rye, millet, teff, quinoa, buckwheat, amaranth)	Meats, legumes, whole grains (gluten-free)	Meats, other legumes, fortified beverages	Meats, seeds, legumes	Meats, legumes, seeds, fortified beverages

Another critical point concerns the patient's education. Clinicians should carefully inform patients and their families regarding what they can eat and provide the appropriate (written or online) resources for additional information (12). Moreover, patients should also be advised on the risk of potential allergen contaminations. According to the specific European legislation (https://www.mise.gov.it/index.php/it/impresa/competitivita-e-nuove-imprese/industria-alimentare/etichettatura-alimentare), clinicians should provide information on packaged foods and educate patients and families to read and correctly interpret the labels of food products. European law established that major food allergens must be declared and reported in the labels of packaged food or available to consumers for non-packaged foods (catering, fresh and cooked foods) (12). Ingredients may change over time, and labels of regularly consumed food should be read each time (12). Fourteen significant allergens must be identified and reported in labels: cereals containing gluten (wheat, rye, barley, oats, spelled, and Kamut), crustaceans, eggs, fish, peanut, soy, milk (including lactose), nuts (almonds, Brazil nuts, cashews, hazelnuts, macadamia, pecan nuts, pistachio nuts, and walnuts), celery, mustard, sesame, sulfites, lupin, and mollusks (https://www.mise.gov.it/index.php/it/impresa/competitivita-e-nuove-imprese/industria-alimentare/etichettatura-alimentare). The precautionary allergen labeling (“may contain”) is not mandatory for European law (12). However, the risk of allergen cross-contamination and trace exposure for foods reporting this warning is variable and still not established in EoE patients (12).

## How to Improve Patient's Compliance: The Role of the Multidisciplinary Team

The chronic nature of EoE, comorbidities, long-term restrictive therapies and strict endoscopic follow-up are the main stressful factors for patients and their families (17). Therefore, it is evident that EoE significantly impacts the QoL of both pediatric and adult patients (17). The complexity and the clinical heterogeneity of this emerging chronic disease implies the need for a multidisciplinary approach, including allergist, pediatrician, gastroenterologist, nutritionist, and psychologist to manage these patients (Table 3) (31). In high specialized Centers, all these specialists should be present during the entire course of the disease and guarantee the transition from the pediatric to the adult setting. Allergists should identify other coexisting atopic comorbidities (eczema, allergic rhinitis, asthma, food/drug allergy, and anaphylaxis) and provide adequate treatment if symptoms are not controlled. Allergy assessment is also fundamental to prevent potential IgE-mediated reactions when foods (especially milk) are reintroduced. Strict clinical and endoscopic follow-up is required to evaluate patient compliance, long-term treatment side effects, and assess disease remission. Notably, children with severe disease, multiple food allergies, non-allergic comorbidities (such as esophageal atresia or genetic disorders) or treated with elimination diets (elemental diets or empirical FEDs) require a regular pediatric evaluation of their growth and nutritional status. Finally, psychological support should be provided when behavioral, mood diseases, or eating disorders are suspected (17).

## Conclusion

EoE is an emerging chronic allergic disease with a relevant impact on the health care system and patients' QoL. Although the pathogenesis is not entirely understood, EoE is a T2 inflammatory disease mainly triggered by food allergens. Diet therapy and medications are both first-line treatments. The choice of one or the other therapy depends on the disease phenotypes (allergic vs. non-allergic, inflammatory vs. fibro-stenotic), patient's age (adult vs. childhood-onset), food habits, patient/family preference, and familiar financial resource. Diet therapy is a successful treatment but limited by low patient adherence, the need for several endoscopies, food restriction, psychosocial impacts, and potential nutritional deficiency. All these limitations could be effectively overcome with multidisciplinary and personalized management. Considering the clinical heterogeneity of EoE, future efforts should be addressed to personalize treatments. Multidisciplinary management, a personalized approach, and proactive education on the importance of treatments and regular endoscopic follow-up may be the keys to a more successful therapeutic strategy.

## Author Contributions

MV reviewed the literature and wrote the manuscript. MDF reviewed the literature and the manuscript. AL, GLM, ADS, MVL, and CMR revised the manuscript critically for important intellectual content. All authors approved the final version of the manuscript and agreed to be accountable for all aspects of the work.

## Conflict of Interest

The authors declare that the research was conducted in the absence of any commercial or financial relationships that could be construed as a potential conflict of interest.

## Publisher's Note

All claims expressed in this article are solely those of the authors and do not necessarily represent those of their affiliated organizations, or those of the publisher, the editors and the reviewers. Any product that may be evaluated in this article, or claim that may be made by its manufacturer, is not guaranteed or endorsed by the publisher.
